# Supplementary material for: Mitochondrial cytochrome b sequence data are not an improvement for species identification in scleractinian corals
Source: PeerJ. 2014 Sep 9;2:e564. doi: 10.7717/peerj.564 (PMC4168843; doi:10.7717/peerj.564)
Supplement: File S2 [file peerj-02-564-s002.pdf]

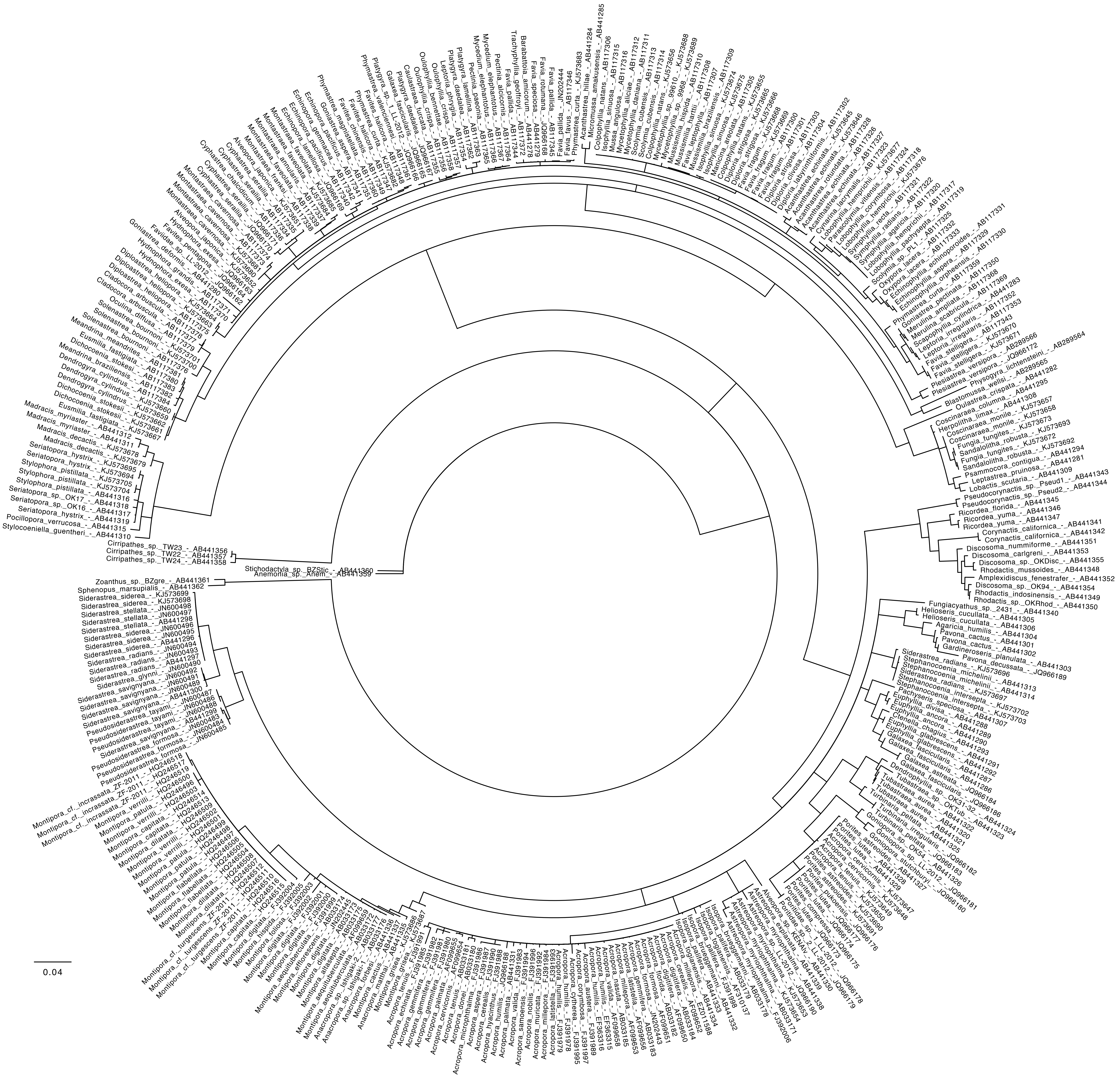

Supplemental Figure S2. Neighbor-joining tree generated using Geneious R7 and the aligned CYB sequence data for corals provided in Supplement S1. Bootstrap support is not shown, but is typically low for most internal nodes (available from JPW). Tree is midpoint-rooted, with no outgroup taxon identified; circular format is provided so all taxon names are visible.
